# Complete mitochondrial genome of *Accipiter trivirgatus*

**DOI:** 10.1080/23802359.2019.1678421

**Published:** 2019-10-18

**Authors:** Fengling Zhang, Lizhi Zhou, Yuanqiu Dong, Yunwei Song

**Affiliations:** a School of Resources and Environmental Engineering, Institute of Biodiversity and Wetland Ecology, Anhui University, Hefei, China;; b Anhui Province Key Laboratory of Wetland Ecosystem Protection and Restoration, Anhui University, Hefei, China;; c Anhui Biodiversity Information Center, Anhui University, Hefei, China;; d Shengjin Lake National Nature Reserve of Anhui Province, Dongzhi, China

**Keywords:** *Accipiter trivirgatus*, mitochondrial genome, gene organization, pseudo-control regions

## Abstract

Crested goshawk (*Accipiter trivirgatus*) is a diurnal raptor tropical Asia which is a bird species in family Accipitridae. In the present study, we determined its complete mitochondrial genome by PCR-based method. The complete mitochondrial genome was 18,454 bp in length which overall base composition was 31.2% A, 24.4% T, 31.0% C, and 13.4% G. It consisted of the typical structure of 13 PCGs, 2 ribosomal RNA (rRNA) genes, 22 transfer RNA (tRNA) genes, and 2 control regions. All of the PCGs started with ATG codon, except for ND3 which was started with ATC. Most of the genes terminate with codons TAA. The non-coding regions include pseudo-control regions.

Crested goshawk (*Accipiter trivirgatus*) is a bird species in family Accipitridae which is a diurnal raptor in tropical Asia. There are 11 subspecies for *Accipiter trivirgatus* complex. Two of them distribute in China, one is *A. t. indicus* in S China and the other is *A. t. formosae* in Taiwan (Dickinson, 2003). Up to date, only partial mitochondrial sequence for *Accipiter trivirgatus* is available. In the present study, we amplified and sequenced the complete mitochondrial DNA of the *Accipiter trivirgatus* for the first time with the hope to provide the basic mitogenome data for future phylogenetic studies of Accipitridae birds.

The muscle sample was collected from a wild *Accipiter trivirgatus* that died of natural causes by accident in Shengjin Lake National Nature Reserve (117° 3′32.59″E, 30°22′48.85″N), Anhui Province, China on January 16, 2019. The specimen is stored at Shengjin Lake Station for Wetland Ecosystem Research (Sample code is SJL20190116). The complete mitogenome sequence of *Accipiter trivirgatus* was amplified and sequenced by the normal LA-PCR and PCR-based methods. The DNA sequence has been deposited in GenBank with accession number MK953813.

The complete mitogenome *Accipiter trivirgatus* was an 18,454 bp circular DNA molecule. The overall nucleotide composition was: 31.2% A, 24.4% T, 31.0% C, and 13.4% G, with a total A + T content of 54.6%.The mitogenome included 13 protein-coding genes, 22 tRNA and 2 rRNA genes, and 2 AT-rich control regions. Of the 13 protein-coding genes (PCGs), the longest one was ND5 (1817 bp), and the shortest was ATP8 (167 bp), similar to the other *Accipiter* species (Song et al. 2015). All of the PCGs started with ATG codon, except for ND3 which was started with ATC. Most of the genes terminated with codons TAA, ND1 and COXI with AGG, ND5 terminated with AGA.COXIII and ND4 genes had an incomplete termination codon “T—”, which was the 5’ terminal of the adjacent gene. The 12S rRNA was 971 bp long and the 16S rRNA was 1610 bp in length. In addition, the non-coding regions included 2 control regions (D-loop).The first D-loop was 1233 bp, located between tRNA^Thr^ and tRNA^Pro^, and secondary D-loop was 1688 bp, located between tRNA^Glu^ and tRNA^Phe^.

Phylogenetic analyses were conducted with mitochondrial genomic data of 14 avian species (including the *Accipiter trivirgatus*) from the GenBank database. The topology of the tree inferred using Neighbour-Joining methods in the programme MEGA 5.05, and bootstrap analysis was performed with 100 replications (Breman et al. 2013). The phylogenetic tree showed that all *Accipiter* species had a close phylogenetic relationship. *Accipiter trivirgatus*, *Accipiter gentilis*, *Accipiter nisus*, *Accipiter soloensis*, *Accipiter virgatus* in one clade, and *Accipiter trivirgatus* had relatively distant systematic relationship to the other four *Accipiter* species (Figure 1).

**Figure 1.**
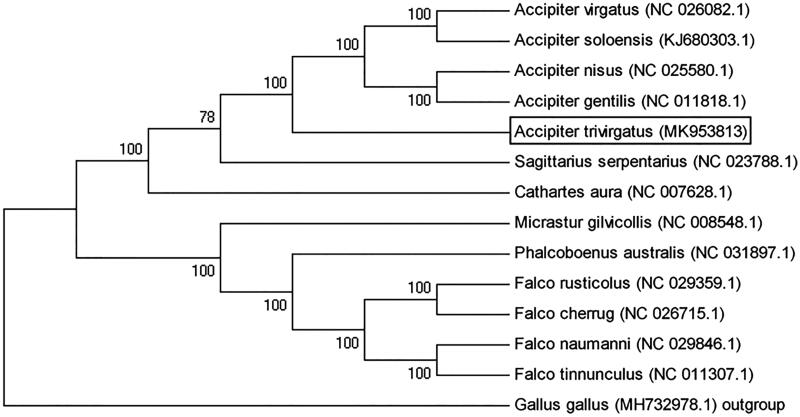
Phylogenetic analysis based on complete mitochondrial genome sequences. An N-J tree were built based on the phylogenetic analysis of 13 Falconiformes species’ complete mitochondrial genomes and *Gallus gallus* used as an outgroup. The mitochondrial genome sequences of analyzed species were obtained from the GenBank databases.
